# News and Coming Events

**Published:** 1907-03-02

**Authors:** 


### NEWS AND COMING EVENTS.

Arrangements are being made for a bazaar to be held in June in aid of the funds of the East London Hospital for Children.

The Ladies' Association of the Shadwell Hospital for Children has decided to endow a Ladies' Association in that institution.

The annual general meeting of the Governors of the Royal Dental Hospital will be held at the hospital on Thursday, March 14, at 5.30 p.m., William H. Ash, Esq., J.P., in the chair.

The festival dinner in aid of the National Hospital for the Paralysed and Epileptic will take place on Monday, March 11, in the Whitehall Rooms of the Hôtel Métropole. Sir Edgar Speyer, Bart., will be in the chair.

The annual meeting of the Royal National Orthopædic Hospital was held on February 27, at which the report of the committee was adopted and the usual routine business was transacted.

Sir Lennox Napier has felt himself obliged to relinquish the chairmanship of the Committee of Management of the West End Hospital for Diseases of the Nervous System, Paralysis, and Epilepsy, but will retain his seat on the Board.

At a recent meeting the Henstead District Council considered the desirability of providing an isolation hospital for the district at Swainsthorpe. It was pointed out that during the last half-year more than £60 had been spent by the Council in dealing with outbreaks of scarlet fever, and that the upkeep of a small isolation hospital would not be very great. Definite action in the matter was finally postponed.

At the annual meeting of the London Fever Hospital the Secretary stated that the increase in the number of patients treated in the hospital was not due to an epidemic, but to a fact which had brought the hospital conspicuously to notice. At the end of 1895 there was a movement in favour of admitting paying patients to the hospitals of the Metropolitan Asylums Board. Thereupon the Secretary of the London Fever Hospital sent a circular to doctors generally pointing out that this institution did such work on reasonable terms, and subsequently the Metropolitan Asylums Board resolved to take no further action in the proposed direction.

The Bradford Eye and Ear Hospital had its origin in a small private dispensary, established by the late Dr. Bronner for the purpose of giving gratuitous advice and treatment to the poor who were suffering from diseases of the eye and ear. The work was at first carried on in two rooms in Rawson Place, adjoining the old green market, and it was watched with so much interest that the private charity was soon converted into a public institution. At this point a house was rented in Brunswick Place, the work being carried on there from 1857 to 1865. The first stone of the present building in Hallfield Road was laid in 1864 by the late Sir Titus Salt, and in the following year the opening ceremony was performed by the late Mr. Charles Semon, then Mayor of Bradford. In the first year there were 610 patients, while last year the number had increased to 7,270, upon whom 24,547 attendances were made.

Lord Armstrong has been elected Chairman of the House Committee of the Royal Victoria Infirmary.

A new workhouse infirmary has been opened at Wetherby. It gives accommodation for 22 patients and has been constructed at a total cost of less than £2,000.

Preparations are being made for an International Medical Congress to be held at Budapest in 1909. The Congress will be divided into twenty sections, and papers may be read in German, English, or French. Many well-known specialists have promised to attend.

The need for a hospital at Ilford has greatly increased since that town became so large a centre of population. For a long time it was felt that it was not proper that in cases of accident and emergency the unfortunate sufferers should have to be taken by road or rail to either the West Ham or the London Hospital. It is probable that the building of Ilford Hospital will soon have been begun. Mr. Holcombe Ingleby gave £1,000 to the fund at a hospital dinner, and a bazaar in aid of the hospital was furnished by nearly all the public organisations in Ilford.

